# Supplementary material for: Modeling glioblastoma heterogeneity as a dynamic network of cell states
Source: Mol Syst Biol. 2021 Sep 16;17(9):e10105. doi: 10.15252/msb.202010105 (PMC8444284; doi:10.15252/msb.202010105)
Supplement: Supplementary file 6 — Source Data for Figure 5 [file MSB-17-e10105-s004.zip › Figure5A_sourcedata/GSEA_3071/nosigdb_stateVI.GseaPreranked.1622795169056/SUVA_MES1_PMID_31327527.html]

Details for gene set SUVA\_MES1\_PMID\_31327527[GSEA]

|  || Dataset | state63071 |
| Phenotype | NoPhenotypeAvailable |
| Upregulated in class | na\_pos |
| GeneSet | SUVA\_MES1\_PMID\_31327527 |
| Enrichment Score (ES) | 0.33924264 |
| Normalized Enrichment Score (NES) | 1.6732692 |
| Nominal p-value | 0.016194332 |
| FDR q-value | 0.034004953 |
| FWER p-Value | 0.327 |
Table: GSEA Results Summary

  

Fig 1: Enrichment plot: SUVA\_MES1\_PMID\_31327527      
 Profile of the Running ES Score & Positions of GeneSet Members on the Rank Ordered List

  

| PROBE | GENE SYMBOL | GENE\_TITLE | RANK IN GENE LIST | RANK METRIC SCORE | RUNNING ES | CORE ENRICHMENT || 1 | SERPINE1 |  |  | 0 | 1.673 | 0.0895 | Yes |
| 2 | MT1E |  |  | 10 | 1.128 | 0.1473 | Yes |
| 3 | MT2A |  |  | 58 | 0.859 | 0.1802 | Yes |
| 4 | ANXA2 |  |  | 79 | 0.783 | 0.2166 | Yes |
| 5 | TUBA1C |  |  | 199 | 0.604 | 0.2159 | Yes |
| 6 | PLP2 |  |  | 205 | 0.600 | 0.2466 | Yes |
| 7 | CLIC1 |  |  | 258 | 0.561 | 0.2622 | Yes |
| 8 | MGST1 |  |  | 298 | 0.543 | 0.2805 | Yes |
| 9 | LGALS1 |  |  | 323 | 0.526 | 0.3020 | Yes |
| 10 | TAGLN2 |  |  | 360 | 0.508 | 0.3192 | Yes |
| 11 | GFPT2 |  |  | 436 | 0.476 | 0.3239 | Yes |
| 12 | CTSB |  |  | 471 | 0.464 | 0.3392 | Yes |
| 13 | EFEMP1 |  |  | 763 | 0.384 | 0.2792 | No |
| 14 | ACTN1 |  |  | 845 | 0.369 | 0.2766 | No |
| 15 | MT1X |  |  | 867 | 0.366 | 0.2903 | No |
| 16 | VIM |  |  | 897 | 0.361 | 0.3016 | No |
| 17 | EMP3 |  |  | 1278 | 0.306 | 0.2128 | No |
| 18 | FN1 |  |  | 1654 | 0.264 | 0.1231 | No |
| 19 | C1R |  |  | 2388 | -0.351 | -0.0610 | No |
| 20 | IFITM3 |  |  | 2851 | -0.477 | -0.1634 | No |
| 21 | GSN |  |  | 3041 | -0.559 | -0.1858 | No |
| 22 | IGFBP7 |  |  | 3428 | -0.958 | -0.2414 | No |
| 23 | A2M |  |  | 3503 | -1.122 | -0.2019 | No |
| 24 | WWTR1 |  |  | 3519 | -1.185 | -0.1427 | No |
| 25 | SPP1 |  |  | 3541 | -1.286 | -0.0797 | No |
| 26 | MGP |  |  | 3612 | -1.988 | 0.0072 | No |
Table: GSEA details [plain text format]

  

Fig 2: SUVA\_MES1\_PMID\_31327527: Random ES distribution      
 Gene set null distribution of ES for **SUVA\_MES1\_PMID\_31327527**
